# An Understanding of Implicit Followership Toward New Employees' Self-Efficacy: The Mediating Role of Perceived Supervisor Support

**DOI:** 10.3389/fpsyg.2021.759920

**Published:** 2021-10-13

**Authors:** Wei Zhang, Xue-Jun Wang

**Affiliations:** School of Economics and Management, Wuhan University, Wuhan, China

**Keywords:** implicit followership, positive implicit followership, negative implicit followership, supervisor support, perceived self-efficacy, workplace friendship

## Abstract

Under turbulent, boundaryless, and Internet age, the characteristics of career sustainability development have shifted from the perspective of development within the organization to the career development track of self-efficacy. New employees usually face the difficult stage of adapting to the new environment and establishing interpersonal relationships with new colleagues. When new employees enter an organization, they usually have different implicit followership cognitions. Previous studies have focused on the treatment of new employees by the organization and the leader, however, the implicit followership cognitive state of new employees has not been studied specifically. This research integrates employees' positive and negative implicit followership, perceived supervisor support, workplace friendship, and perceived self-efficacy into a research framework. This study used a questionnaire survey by an online professional survey website. A total of 394 valid questionnaires were collected. Structural equation model (SEM) analysis was carried out and according to the results, new employees' positive and negative implicit followership significantly affects perceived supervisor support. Furthermore, perceived supervisor support had a significant impact on perceived self-efficacy. Moreover, perceived supervisor support was found in a mediating role between the relationship of implicit followership theories and perceived self-efficacy. Finally, workplace friendship was found to be a significant moderator in the relationship between perceived supervisor support and perceived self-efficacy. Based on the research results, business managers are suggested to pay more attention to new employees' self-cognition of their job roles and enhance the self-efficacy of new employees in the entry stage.

## Introduction

Under turbulent, boundaryless, and Internet age, new generation employers are different from the traditional concepts of the past generations. On the professional track, the characteristics of career sustainability development have shifted from the perspective of development within the organization to the development of self-efficacy. For new employees, self-efficacy perception can be regarded as employees' positive psychological resources, it can motivate individuals to work. How to enhance employees' self-efficacy is an important key to career sustainability development. According to Bargh's research (Bargh, 1997), implicit processes such as habits, schemas, and intuitions will shape individual behavior and attitudes in the workplace, which means that employees' cognition–implicit followership will affect self-efficacy.

Furthermore, the implicit followership theory suggests that people create individual beliefs regarding the qualities that characterize followers. These perceptions are kept in the mind as followership prototypes and are triggered when individuals cooperate with actual followers (Wang and Peng, 2016). Follower-focused leadership scholars have shown that the follower viewpoint enriches considerably to the knowledge of leader understanding and conduct, the methodology utilized by leaders to administer information—specifically, their view of followers—and the establishment of leadership. Though, for the investigation to be further significant, research on followership from the viewpoint of the employee must be centered on the widespread categorization of key tendency models or goal-oriented standard models (Gao and Wu, 2019). According to studies, different leaders can build various implicit models of followers (Shondrick et al., 2010). Implicit followership theory (IFT) is centered on the concept of social cognition that gives extra consideration to the cognitive formation of different followers. Based on the cognitive classification model, the leader will engage in comparisons between explicit followerships of the team associates, once the leader's IFT is triggered. This comparison will be used to create a consequent theoretical cognition of the team participants and implement the consequent behavioral framework corresponding to this cognition (Afzal et al., 2019; Wang and Liang, 2020). These queries have been tackled by scholars in the current development of IFT (Shondrick et al., 2010; Sy, 2010; Epitropaki et al., 2013; Junker and van Dick, 2014; Uhl-Bien et al., 2014; Junker et al., 2016) that includes individuals' beliefs regarding the qualities and actions that describe followers (Sy, 2010). Research scholars discovered that implicit followership theory includes positive as well as negative elements (Wang and Liang, 2020). In this study, we have treated IFT positive dimensions as enthusiasm, good citizen, and industry whereas, negative dimensions as inactive and disobedience.

Moreover, in terms of leadership theories, some connections have been found between implicit followership theory and perceived supervisor support. Perceived supervisor support (PSS) has obtained immense interest from researchers as it influences individuals' job results (Gentry et al., 2007; Chen and Chiu, 2008). PSS develops self-confidence in workers (Pan et al., 2011) regarding their skills to carry out their duties at the organization (Silbert, 2005). In associating implicit followership theories with followers' attitudinal and behavioral consequences in earlier investigations (Sy, 2010), the similarity between implicit followership theories and views of real followers is generally accepted; though, the similarity is certainly not immediately verified. Consequently, these researches disregarded the leaders' opinions of followers relating to implicit followership theories in producing attitudinal and behavioral results (Goswami et al., 2020). Researchers primarily utilize implicit followership theory in the method of business management, and their study concentrates on the structure and the impact of implicit followership theory (Wang and Liang, 2020). Corresponding to the Pygmalion effect, the leader has great expectations and encourages and supports those individuals that are engaged in the model that they are following positively (Whiteley et al., 2012). This commendable impact is likewise recognized by other team representatives that achieve the knowledge of substitution. In this method, the positive physiological and emotive knowledge of the followers is steadily stimulated and constantly built toward the model of the follower anticipated by the leader (Hoption et al., 2015). The team leader's forming of the positive follower model supports the team participants try to attain the qualities and conducts of the positive followers to accomplish success in careers more certainly. Implicit followership theory is a novel concept that uncovers the psychological system of leadership that was created by western contemporary management scholars based on social cognition theory. Following the advent of this theory, it has been studied and accepted by researchers around the globe. Nevertheless, researchers have not established a comprehensive hypothetical model for the research of implicit followership theory. The goal of the present research is to fulfill this gap by precisely analyzing the association between implicit followership theories and perceived supervisor support. The IFT dimension of the congruence also matters, hence, this research hypothesized that the association between both positive and negative IFTs and perceived supervisor support will cause various outcomes.

In addition to perceived supervisor support, this research also incorporated self-efficacy which is based on a similar cognitive motivation idea and is related to the fundamental notion of social cognition theory that was originally projected by Bandura (1977). Corresponding to this notion, self-efficacy is a principle of a person's cognition that primarily indicates a person's view and belief in effectively completing a particular job or several jobs (Bandura, 1977) in addition it can influence a person's assessment, choice, and motivation (Tierney and Farmer, 2002). The theory of self-efficacy additionally states that experience modifies psychology and conduct via self-control, and the option of conduct improves the stability of associated behaviors. This theory combines the emotion and cognition of an individual. Initiating from the intellectual aspects, the social motivation of the person is examined. Bandura (1982) felt that the cause of self-efficacy principally comprises four dimensions—self-efficacy experience, emotional state, verbal encouragement, and, alternate experience. Social learning theory (Bandura, 1971) might be useful to clarify the association of perceived supervisor support with self-efficacy. Bandura (1977) has delivered important efforts concerning self-efficacy and social learning theory. Bandura indicates that individuals understand from experiences and build their self-efficacy. At an organization, the self-efficacy of personnel can be created once they are operating in a positive working environment. These positive circumstances can be offered in the shape of perceived supervisor support (Afzal et al., 2019). Self-efficacy inspires a determination of self-belief between the workforce, therefore, they are expected to remain on the job and confront the challenges. Hence, this research explores the association between perceived supervisor support and self-efficacy. Furthermore, this study also proposes and analyzes the indirect relationship of IFT and self-efficacy through the mediation of PSS.

This research aims to examine the differences in perceived supervisor support of new employees that how to be affected by the employee's own implicit followership cognition, and the relationship between this perception and the employee's perceived self-efficacy. This research breaks through the limitations of previous research that focused on positive IFT. In this study, positive and negative implicit followership theories are analyzed as separate dimensions of the followers' conduct. The negative aspect of implicit followership theories has not been studied considerably in the past, but they are very crucial theoretically. For instance, negative experiences and feelings tend to inflict greater impacts on individuals around a wide variety of concerns (Goswami et al., 2020). Due to the bad-is-stronger-than-good principle, it is remarkably essential to investigate negative as well as positive implicit followership theories. From the two dimensions of follower's positive and negative IFTs, we explore how the difference in employee perception affects their perceived supervisor support. In addition, according to the theory of social relations, the formation of one kind of social relationship will have an impact on another kind of social relationship (Tse et al., 2008). Therefore, this study introduced workplace friendship to explore the moderation effect of workplace friendship between perceived supervisor support and perceived self-efficacy. This research aims to examine at least four research gaps. First, it analyzes the impact of IFT on PSS. Second, it examines the influence of PSS on self-efficacy. Third, it investigates the indirect mediation effects of PSS on the association between IFT and self-efficacy. Finally, it explores the moderation effect of workplace friendship on the relationship of PSS and PSE. This study attempts to explore new employees' self-efficacy from the perspective of followers' implicit followership. Effective onboarding can increase benefits by hiring talented employees and increase the utilization of the hard work spent in recruiting and selecting these employees. This research mainly discusses the implicit follower of new employees and explores the results of its impacts. Based on the theory of social exchange and social relations, combined with self-efficacy, and perceived supervisory support, this study puts forward the research framework and related hypotheses.

## Background and Hypotheses Development

### Implicit Followership Theory and Perceived Supervisor Support

The Implicit Followership Theories (IFTs) were first proposed by Thomas Sy in 2010 (Sy, 2010). In the past 30 years, the theoretical circle has established a rich system of Implicit Leadership Theory (ILT) but there is very little research on implicit followership theories. As the leadership field gradually stressed the significance of followers, ILT research was extended to IFTs (Lord et al., 2020). In an organizational environment, individuals naturally tend to classify people as leaders or followers. Therefore, Sy puts forward the concept of opposite to Implicit leadership—Implicit followership and believes that implicit followership is the schema and belief of the traits and behavior of followers (employees) (Sy, 2010). The theory is mainly derived from implicit theory (Greenwald and Banaji, 1995). That is, over time, based on long-term accumulated experience, individuals will form a preconceived cognitive model for the behaviors and characteristics of the role (leader or employee) in the organization (Shondrick et al., 2010). Explaining the role of employees from the perspective of implicit followership, a cognitive schema is an implicit cognition of the followership, which cannot be discovered at the conscious level. It is also the essence of implicit followership theory (Jian and Xiao, 2015). This implicit cognition may affect individual judgment and behavior.

According to the contents and dimensions of “expected followership,” the followership cognitive schema is divided into positive and negative implicit followership. Positive implicit followership is a cognitive composition about the positive characteristics of followers. That is a series of expected follower traits and abstract representations of behavior in the mind, such as diligence, enthusiasm, and good citizenship. On the other hand, the negative implicit follower identity is the cognitive structure of the negative features of the follower. In the mind of abstract representation, this is a series of follower characteristics and behaviors, such as incompetence, submission, and rebellion. Since implicit followership is not as easy to be observed as explicit behavior, there are some controversies in the measurement of implicit followership (Epitropaki et al., 2013).

PSS refers to the employees' overall perception of the supervisor's attention to employees' contribution and concern for their happiness (Kottke and Sharafinski, 1988). The characteristics and actions exemplified in implicit followership theories aid the leader to propose judgments regarding a principal follower. Furthermore, positive or negative attributes exhibited will improve or reduce the influence of the dyadic partner in the affiliation, once corresponding with individual Implicit followership theory. A study portraying somewhat similar findings by Engle and Lord (1997) showed that supervisors usually utilize implicit theories to decide the value of leader-member exchange. This research paper explicitly indicates that the more the main follower displays positive characteristics and activities created in the leader's positive implicit followership theories, the more it is expected that the leader will positively assess their impact to the association (Sy, 2010; van Gils et al., 2010).

On the other hand, abused workers account for low life and job satisfaction (Tepper, 2000; Zellars et al., 2002), and, greater supervisor- and company-produced eccentricity (Tepper et al., 2009). Negative implicit followership theories and negative views of a follower would cause adverse leader actions for instance abusive supervision (Shondrick et al., 2010). In other words, similarity among leader's negative implicit followership theories and their adverse views of a follower must extract adverse supervisory activities. In Particular, negative follower actions and traits such as being impolite and egotistical are extremely prone to be compatible with negative implicit followership theories. Moreover, if the negative implicit followership theories of a leader meet with workers at high results contrasted to if a leader's negative implicit followership theories meet with workers at low results, the leader will treat this follower further adversely (Goswami et al., 2020).

Regarding the dimensions of perceived superiors' support, there are different opinions in the academic circles. First, the scholars that hold a one-dimensional view regarding the perceived supervisory support as an independent concept (Kottke and Sharafinski, 1988). Then, scholars who hold a multi-dimensional view will divide the perceived supervisor support into emotional support and instrumental support (Amabile et al., 2004). Moreover, some scholars argued that PSS is one of the dimensions of perceived organizational support. It can be divided into task-oriented support and relationship-oriented support (Organ et al., 2005). Last, this research mainly focuses on employees' perception of supervisor's support. Therefore, this study adopts a single-dimensional view of perceived supervisor support.

Implicit followership is an individual cognition. Studies have shown that individual cognition affects the perceived supervisor support (Rui and Wenquan, 2008). Therefore, employees will also perceive higher levels of supervisor support. Conversely, employees with negative implicit followership will affect their perceived supervisor support. New employees usually lack experience and abilities. They are more sensitive to the organizational environment and the behavior of their supervisors. Based on the above discussion, the research hypothesis can be proposed as follows.

H1: The positive implicit followership of new employees has a positive significant impact on perceived supervisor support.
H2: The negative implicit followership of new employees has a negative significant impact on perceived supervisor support.

### Perceived Supervisor Support and Self-Efficacy

Self-efficacy is a person's belief, judgment, or overall control and attitude about the ability to complete an activity (Bandura, 1977). People's motivation, emotional state, and level of action are more based on their beliefs, rather than objectively based on facts. Therefore, self-efficacy is the basic prerequisite for individuals to make behaviors suitable for their environment (Hsu et al., 2007). Employees with a high level of perceived self-efficacy are more confident in controlling the external environment. It also can promote individual initiative and facilitates the completion of work (Bandura, 1977).

Individual self-efficacy can be enhanced through successful experience, verbal encouragement, and emotional support (Bandura, 1977). Studies have shown that employees' feedback-seeking has a substantial constructive effect on self-efficacy. Self-efficacy is found to be positively associated with psychological and physical welfare. Moreover, supervisor support as a kind of situational support is also an important source of employees' self-efficacy. Once employees have a high degree of PSS, they can focus on the work process. Thereby, supervisor support would reduce the anxiety and work pressure of employees. Moreover, the self-efficacy of employees will be improved (Walumbwa et al., 2011). Ashforth and Saks (2000) discovered that people greater in self-efficacy engaged with complicated circumstances with a problem-centered method. It reduced their situation of susceptibility and directed them to greater dedication and job participation. Workers possessing high levels of self-efficacy are likely to react to adverse feedback with a rise in work and enthusiasm. Consequently, they are inclined to remain at their jobs and outshine instead of thinking about leaving the employment (Afzal et al., 2019).

Tschannen-Moran and Hoy (2007) argued on teachers' self-efficacy that, control experience is assumed to be the most powerful source. For individuals who have less experience, other sources of self-efficacy will play a greater role in early learning. This research survey of novice teachers and in-service teachers found that contextual factors such as important interpersonal support have a more significant impact on the self-efficacy beliefs of novice teachers. Among experienced teachers, contextual factors such as interpersonal support have a more significant influence on their self-efficacy beliefs. The impact is much smaller. Furthermore, Afzal et al. (2019) surveyed academic staff in colleges and universities and found that the supervisor's sense of support can enhance employees' self-efficacy and further improve their work performance.

In summary, because new employees are relatively unfamiliar with the new working environment, and the content of work is also due to the differentiation of organizational skills, the employees have an insufficient sense of the new job experience. The supervisor usually acts as the agent of the organization and is in a better position to make formal decisions concerning resource allocation and setting priorities between tasks (Škerlavaj et al., 2014). Therefore, this study believes that the supervisor's sense of support will significantly promote the impact of new employees' self-efficacy in this process. Based on the above discussion, the following hypotheses are proposed:

H3: New employees' perceived supervisor support will positively and significantly affect employees' perceived self-efficacy.

### Mediating Effect of Perceived Supervisor Support on IFT and Self-Efficacy

Implicit followership theories are psychologically intellectualized illustrations and beliefs related to the followers. These beliefs become encrypted as cognitive types and are collected in the memory of the leader (Rosenberg and Jones, 1972; Sternberg, 1985). Additionally, positive IFTs represent beliefs of constructive characteristics and activities of followers including good citizen, industry, and enthusiasm, whereas negative IFTs are negative psychological prospects of leaders, like believing followers to be inactive and disobedient (Sy, 2010). Research suggests that the kind of belief held, or psychological model supported influences the people to react in a way coherent with that belief (Lord et al., 1984; Engle and Lord, 1997). These characteristics and activities are collected as a consequence of leaders' previous experiences to produce their implicit followership theories. These implicit followership theories manipulate the leaders' evaluation and reaction to their existing followers (Goswami et al., 2020). Great connections among leaders and followers grow throughout time by both sides making efforts that are valued by the other side. Implicit followership theories refer to those significant contributions anticipated from a worker, in general. Once the workforce is presumed to drop short of this standard by the perception of their supervisor, the workforce will believe their supervisors to participate less, implying a low-quality affiliation. Followers must, nevertheless, believe their leaders' view of the perfect follower to be similar for all the workforce. Consistent with the overall notion of implicit followership and leadership theories (Sy, 2010; van Gils et al., 2010), it can be believed that cognitive representations of the perfect follower are invariant around a range of employees. This idea is tacitly made in a lot of the studies on implicit followership theories, stating that only the actual workers are graded on IFT qualities (Braun et al., 2017).

Since IFTs were proposed, researchers have found that the perception-behavior link explains the theory well, assuming that the activation of the implicit followership schema leads to behavior consistent with the schema (Lord et al., 2020). Research on the Pygmalion effect or self-fulfilling prophecy of IFTs proves the perception-behavior link (Whiteley et al., 2012). According to Bandura (Bandura, 1982), the leader of an organizational team, who is striving to achieve a positive prototype will motivate the individuals in his team to adopt the behaviors of positive followers. As per the Pygmalion effect, the leader will provide support to the prospective followers, if the followers are found to be in correspondence with the positive prototype's based conducts (Whiteley et al., 2012). The leaders belonging to the scientific study teams of a university are more likely to create a comparatively strong teacher-student relation by psychologically following the specific fondness of the efficient person via the prototype theory of positive followership in mind. Once implicit followership theories are formed and triggered, the followers understand the belief and support from the leader (Wang and Liang, 2020). This study adopts the employee perspective to empirically how the IFTs dimension plays a role in the self-actualization effect, especially for new employees. This kind of employee does not yet have a complete sense of control over their work experience in the new workplace environment.

Perceived supervisor support can be a possible cause of self-efficacy. Tschannen-Moran and Hoy (2007) asserted that experiences are a powerful resource of self-efficacy, whereas for newcomers organizational sources and interactive assistance as crucial factors of self-efficacy. Current researches support that a decent link with advisors can supplement self-efficacy (Day and Allen, 2004). In managerial situations, efficient supervision is believed as one of the developing and persuasive sources (Oentoro et al., 2016). Supervisors have extensive expertise, and they are properly informed of the demands of subordinates. Moreover, they have a high understanding of managerial situations and are responsible for subordinates' advancement and performance (Pan et al., 2011). Moreover, innovative abilities and expertise are assigned by supervisors to their workers (Lankau and Scandura, 2002). In everyday life, personnel usually obtain guidance from their supervisor. Bandura (1982) recommends four factors that boost self-efficacy: enactive command, graphic patterning, verbal influence, and stimulation. Out of these aspects, verbal influence is particularly pertinent to PSS. A helpful supervisor holds a belief in the skills of the subordinate and can convey this belief in the shape of vocally communicating faith, admiration, and belief (Tierney and Farmer, 2002). Thus, the supervisor can persuade the workers of their skills to accomplish the designated objectives. These opinions are prominent in producing values of self-efficacy in the workers. Consequently, perceived supervisor support significantly influences the self-efficacy of workers (Gibson et al., 2009). It is recommended here that the greater degrees of self-efficacy formed via supervisor assistance can assist employees to establish lasting reactions and have a powerful impact on job fulfillment, dedication, managing activities, and retirement intuitions (Gruman et al., 2006).

The above assessment suggests that the belief from essential others in the corporation makes the specific self-cognition that in turn affects the person's conduct. Consequently, in this research, the PSS is deemed as the mediating variable between the implicit followership theories and self-efficacy.

H4: The perceived supervisor support has a mediating effect between the positive implicit followership of new employees and self-efficacy.
H5: The perceived supervisor support has a mediating effect between the negative implicit followership of new employees and self-efficacy.

### Workplace Friendship

Workplace friendship is the concrete manifestation of friendship in the workplace. It is the informal interpersonal relationship that people form in the workplace. Berman et al. defined it as “a non-exclusive workplace relationship that includes mutual trust, commitment, reciprocal preferences, and shared interests or values” (Berman et al., 2002). The concept of workplace friendship first appeared in 1971. Psychologists put friendship opportunity as one of the elements of the job characteristic model (Hackman and Lawler, 1971). In 1995, researchers turned their research perspective to the intensity and quality of friendship in the workplace. They proposed the concept of friendship quality, which further advanced the research on workplace friendship (Riordan and Griffeth, 1995). Furthermore, Nielsen et al. (2000) created a workplace friendship scale based on friendship opportunities and friendship intensity. The development and improvement of the scale laid a framework for discussing workplace friendships in academic research. From an individual perspective, as an informal interpersonal relationship in the workplace, workplace friendship will promote mutual exchanges between colleagues, enhance trust among employees and provide mutual support. Kram and Isabella (1985) elaborated on the function of colleague friendship more systematically and believed that workforce friendship can promote individual career development. However, the negative effects of workplace friendships also exist. Compared with individuals in colleague relationships, individuals with workplace friendships are more likely to experience social and emotional interference from frequent social interactions, which in turn interferes with their investment in instrumental goals (Pillemer and Rothbard, 2018).

Specifically, workplace friendship is an intimate partnership between colleagues developed based on formal work contacts, with non-exclusive, informal, and personal characteristics. With a high level of workplace friendship, employees can get more supportive social resources (Liu et al., 2013). Individuals with low workplace friendships reflect that the quality of relationships between individuals and colleagues around them is poor, and they are more likely to face social pressure from colleagues (Kui et al., 2018).

Workplace friendship is an informal relationship where partners invest time and effort to build friendships. It would bring emotional satisfaction and instrumental support to each other. Based on the social exchange theory, an individual's certain exchange relations will affect the formation of other exchange relations. Therefore, this study believes that workplace friendship will moderate the connection between perceived supervisor support and self-efficacy. Workplace friendship has the nature of two-way information flow (Mao et al., 2012). Work-related problems are usually one of the situational factors that cause workplace friendships (Sias et al., 2003). When employees have a high level of workplace friendship, adequate information exchange from colleagues can help employees reduce their anxiety about uncertainty and challenging work. Then, it can produce a more positive work mentality under the perceived supervisor's support, and stimulate its self-efficacy. When employees have low-level workplace friendships, employees are in a relatively isolated state among colleagues, which increases their perception of risks and costs (Cao and Zhang, 2020). This counteracts the positive emotional response brought by the support from the supervisor and reduces their sense of self-efficacy. Based on this, this article proposes the following hypotheses:

H6: Workplace friendship has a significant moderating effect between new employees' perceived supervisor support and self-efficacy.

**Figure 1:**
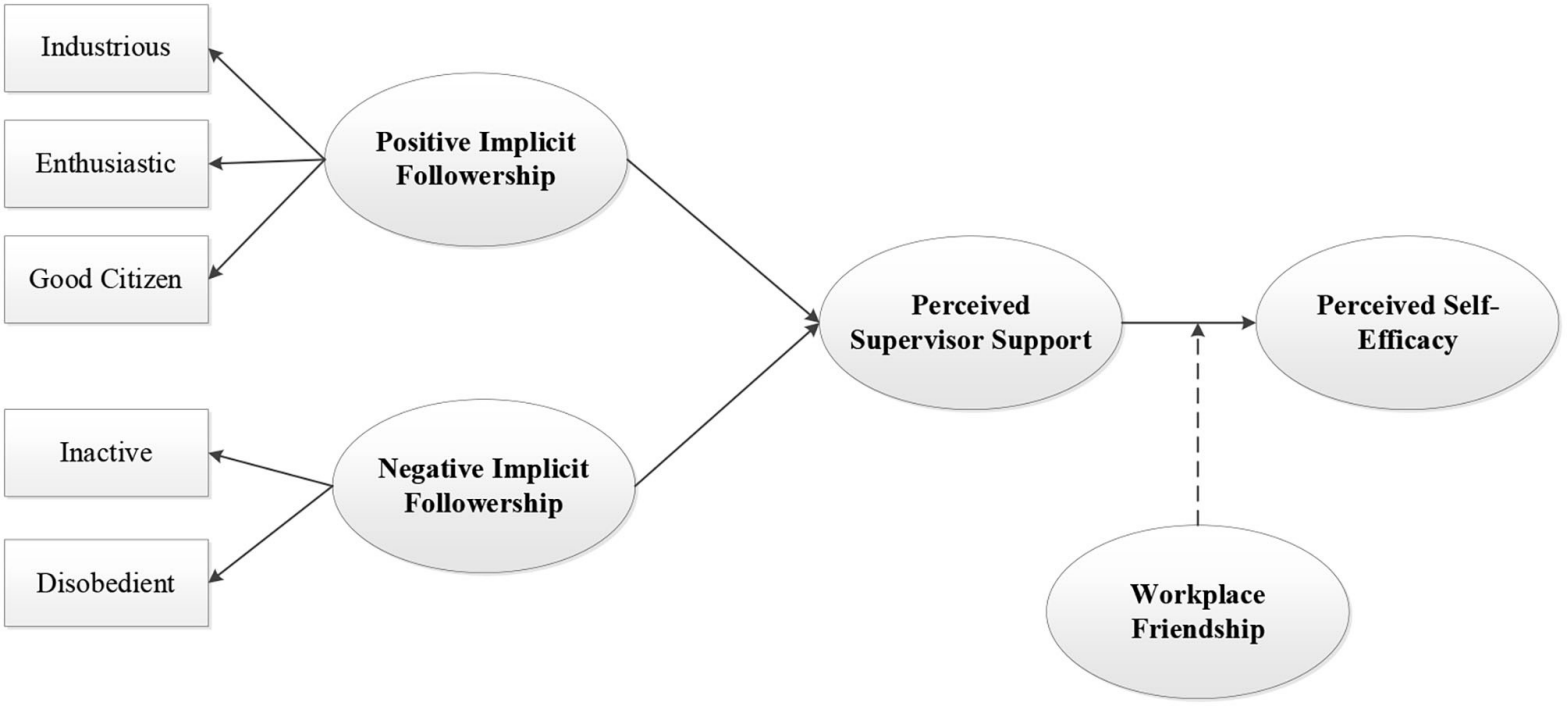
Theoretical framework.

According to the above hypotheses development, we proposed our research model in Figure 1.

## Materials and Methods

### Data Collection

This research mainly takes new employees as the representative for the sample of this study. New employees are defined as employees staying at an organization for <3 years. The data in this questionnaire survey was collected by using the method of snowballing convenience sampling. The questionnaires were distributed to qualified recruits around team members, and these employees were then sent to the recruits around them for collection. Some of the data were collected by contacting the corporation's human resources manager. The staff of the Resources Department conducted the questionnaire survey to the company's new employees. Most of the survey samples were in various regions of Guangdong Province, and a few samples were distributed in other provinces. The questionnaire collection time was from November 20, 2020, to the end of February 2021. A total of 394 questionnaires were collected, after removing invalid questionnaires with a statistical confidence of 95%, the sample size of the total population was 384 copies. Hence, the sample size of this study was adequate.

### Measurement Instrument

The questionnaire contained the review of the fundamental participants' background data and their views of the study constructs. The background data, containing gender, education, and age was examined in the first section of the questionnaire. In the second section of the questionnaire, the latent variables were calculated by a Likert seven-point scale ranging from “strongly disagree” (1) to “strongly agree” (7). The measurement elements were primarily modified from the earlier experiments. All items were initially written in Chinese and adjusted for the survey participants.

First, there were nine items related to newcomers' positive implicit followership and six items related to negative implicit followership. All 15th items were adapted and modified from Sy (2010), whose scale has good applicability in China. After that, there were six items about perceived supervisor support comprising two items from Kottke et al.'s (Kottke and Sharafinski, 1988) research, and four items from Oldham and Cummings's study (Oldham and Cummings, 1996). Then, perceived self-efficacy was also evaluated by 10 items. All items were adapted from Schwarzer et al. (1997). This scale is a revised scale after comparing the German, Spanish and Chinese versions of the General Self-Efficacy Scale by Schwarzer et al.

### Data Analysis

The findings were handled in two sections comprising measurement model verification, and structural equation modeling (SEM) analysis to achieve the appropriate conclusions. Anderson and Gerbing's (1988) methodology was used to verify the measurement model by confirmatory reliability analysis, convergent validity, and discriminant validity. Subsequently, based on the research model, the structural equation model was analyzed, including path analysis and mediation effect analysis by statistical software AMOS (SPSS Inc., Chicago, IL, USA).

## Results

### Descriptive Statistical Analysis

#### Frequency Distribution

The categorical data elements of the 394 valid questionnaires included gender, education, length of service, and first job. The respondents were 27.2% female. Regarding the education level, the largest group was college and university graduates, which was 93.2%. A total of 233 respondents were at their first jobs, which was 59.1%. The data are shown in Table 1.

**Table 1:** Frequency distribution table.

**Variable**	**Value label**	**Frequency**	**Percent**
Gender	Male	287	72.8
	Female	107	27.2
Education	College or below	215	54.6
	University	152	38.6
	Master or above	27	6.9
Length of Service	3 months or below	75	19.0
	4–6 months	63	16.0
	7–9 months	42	10.7
	10–12 months	21	5.3
	12–36 months	193	49.0
First Job	Yes	233	59.1
	No	161	40.9

#### Item Statistical Analysis

Table 2 shows the mean values and standard deviation values of items associated with each construct. The lowest average score of all items was 2.954, which was for the measurement item stating “I don't seek additional training or experience” of the negative implicit followership construct. On the other hand, the highest average score was 6.348, which was related to the measurement item stating “I am socialized and friendly with others” of the positive implicit followership. The lowest standard deviation value of all the items was 0.756 and this item was related to the positive implicit followership construct.

**Table 2:** Mean and standard deviation of items.

**Construct**	**Item**	**Mean**	**SD**
Positive implicit followership	1. I am continuing to work hard until the work is done.	6.200	0.941
	2. I can get all of my work done.	6.046	0.907
	3. I can engage in additional work that is not expected from the company.	5.513	1.196
	4. I usually show a lot of excitement.	5.680	1.221
	5. I am socialized and friendly with others.	6.348	0.787
	6. I usually smile, and express positive spirits.	6.183	0.904
	7. I support the rules and regulations of my organization.	6.109	1.029
	8. I consistently get my work done on time.	6.119	0.896
	9. I collaborate well with others.	6.244	0.756
Negative implicit followership	1. I am easily express overconfidence.	3.444	1.637
	2. Because of my personality, sometimes I would be impolite or disrespectful.	3.338	1.862
	3. I usually disagree with others.	3.320	1.727
	4. I would not search for knowledge proactively about the work.	2.881	1.691
	5. I often insist on my opinion.	3.964	2.044
	6. I am not very interested in additional training or experience at work.	2.954	1.726
Perceived supervisor support	1. My supervisor will help employees solve work-related problems.	5.789	1.200
	2. My supervisor encourages employees to develop new skills.	5.954	1.030
	3. My supervisor will praise good work performance.	5.891	1.101
	4. My supervisor cares about employees' feelings and thoughts.	5.779	1.242
	5. My supervisor cares about my overall satisfaction with the job.	5.794	1.137
	6. My supervisor will seriously consider my goals and values.	5.726	1.222
Self-efficacy	1. I can always manage to solve difficult problems if I try hard enough.	5.721	1.189
	2. Even if someone opposes me, I can find means and ways to get what I want.	4.746	1.480
	3. It is easy for me to insist on my aims and accomplish my goals.	4.607	1.553
	4. I am confident that I could deal efficiently with unexpected events.	5.114	1.233
	5. With my wisdom, I can deal with emergencies.	4.860	1.245
	6. If I make the necessary efforts, I will be able to solve most problems.	5.454	1.107
	7. I can face difficulties calmly because I trust my ability to deal with problems.	5.383	1.136
	8. When I am confronted with a problem, I can usually find several solutions.	5.343	1.057
	9. When in trouble, I can usually think of some ways to deal with it.	5.452	1.036
	10. No matter what comes my way, I'm usually able to handle it.	4.997	1.201
Work friendship	1. In my organization, I have the chance to talk informally and visit with others.	5.211	1.276
	2. I can work with my coworkers to collectively solve problems.	6.033	0.857
	3. I have the opportunity to get to know my coworkers.	5.840	1.010
	4. Communication among employees is encouraged by my organization.	5.904	0.981
	5. As long as the tasks are completed, my organization allows informal conversations.	5.317	1.267
	6. Being able to see my coworkers is one reason why I look forward to my job.	5.226	1.310
	7. I think I can trust many colleagues.	5.487	1.219
	8. I have the opportunity to develop close friendships at my workplace.	5.591	1.160
	9. I can confide in people at work.	5.591	1.118

### Convergent and Discriminant Validity

This study assessed the measurement and structural model adopting the two-phase method of SEM proposed by Anderson and Gerbing (1988). The first step used Confirmatory Factor Analysis (CFA) to examine the construct convergent validity and reliability of the measurement model. Additionally, in the first phase of SEM analysis, the discriminant validity of the measurement model was also analyzed. Furthermore, maximum likelihood estimation (MLE) was implemented to calculate the factor loadings, convergent validity, and discriminant validity. Then, the second step tested the path effects and their significance of the structural model.

Fornell and Larcker (1981) recommended three indexes for measuring the convergent validity of the measurement elements. The first one is the measurement of item reliability, whereas, the second one is to compute the constructs' composite reliability (CR), the last step is to analyze the average variance extracted (AVE). In a construct, composite reliability implies the internal reliability of each indicator. Items that were not at the threshold level were removed. Table 3 shows, the standardized factor loadings of items ranging between 0.624 and 0.942, indicating all the items fall into a reasonable range and have convergent validity. All the CR of the constructs ranges between 0.831 and 0.938, hence exceeding the 0.6 thresholds recommended by Fornell and Larcker (1981) indicating that all constructs have internal consistency. Lastly, all AVE ranging from 0.584 to 0.717, exceed 0.5 suggested by Hair (1998) and Fornell and Larcker (1981). All constructs possess sufficient convergent validity.

**Table 3:** Results for the measurement model.

**Construct**	**Item**	**Factor loadings**	**Composite reliability**	**Average variance extracted**
Positive implicit followership	PIF 1	0.865	0.848	0.655
	PIF 2	0.942		
	PIF 3	0.654		
	PIF 4	0.624		
	PIF 5	0.877		
	PIF 6	0.865		
	PIF 7	0.770		
	PIF 8	0.856		
	PIF 9	0.775		
Negative implicit followership	NIF 1	0.704	0.831	0.624
	NIF 2	0.799		
	NIF 3	0.865		
	NIF 4	0.924		
	NIF 5	0.672		
	NIF 6	0.747		
Perceived supervisor support	PSS 1	0.729	0.938	0.717
	PSS 2	0.794		
	PSS 3	0.885		
	PSS 4	0.908		
	PSS 5	0.886		
	PSS 6	0.865		
Perceived Self-efficacy	PSE 1	0.713	0.933	0.584
	PSE 2	0.627		
	PSE 3	0.673		
	PSE 4	0.857		
	PSE 5	0.797		
	PSE 6	0.766		
	PSE 7	0.816		
	PSE 8	0.805		
	PSE 9	0.751		
	PSE 10	0.805		

*PIF, Positive implicit followership; NIF, Negative implicit followership; PSS, Perceived supervisor support; PSE, Self-efficacy*.

Contrasting the square root of the AVE of a provided construct with the correlations between the construct and the other constructs is the discriminant validity (Fornell and Larcker, 1981). The indicators are further strongly associated with the construct than the others if the square root of the AVE of a construct is higher than the off-diagonal elements in the corresponding rows and columns. As in Table 4, the bold numbers in the diagonal direction represent the square roots of AVEs. Because all the numbers in the diagonal direction are greater than the off-diagonal numbers, discriminant validity appears to be satisfactory for all constructs.

**Table 4:** The result of discriminant validity analysis.

**Construct**	**AVE**	**PSS**	**PSE**	**PIF**	**NIF**
PSS	0.717	**0.847**			
PSE	0.584	0.431	**0.764**		
PIF	0.779	0.457	0.197	**0.883**	
NIF	0.751	−0.405	−0.175	−0.224	**0.867**

*The diagonal elements in bold is the square root of AVE. PSS, Perceived supervisor support; PSE, Self-efficacy; PIF, Positive implicit followership; NIF, Negative implicit followership*.

The present study implemented eight common models of fit verification methods proposed by Jackson, Gillaspy, and Purc-Stephenson (Jackson et al., 2009). Moreover, if the sample size is larger than 200, the Chi-square value can get an insignificant outcome. Hence, the bootstrap method provides an alternative way to get a better result. By using Chi-square divided by degree of freedom (DF), the ideal result should be <3. Furthermore, other criteria provide a more rigorous standard for model fit verification, as shown in Table 5. For instance, the Root Mean Square Error of Approximation's (RMSEA) value should be <0.08, whereas, Comparative Fit Index (CFI) criteria should be higher than 0.9. The tested results are shown in Table 5. All the model fit criteria tested fitted the suggested standards.

**Table 5:** Model fit verification.

**Model fit**	**Criteria**	**Model fit of the research model**
χ^2^	The small the better	558.438
DF	The large the better	425
Normed Chi-sqr (χ^2^/DF)	1< χ^2^/DF<3	1.315
RMSEA	<0.08	0.028
TLI (NNFI)	>0.9	0.983
CFI	>0.9	0.984
GFI	>0.9	0.938
AGFI	>0.9	0.925

*χ^2^, Chi-square; DF, Degree of Freedom; RMSEA, Root Mean Square Error of Approximation; TLI (NNFI), Tucker-Lewis Index (Non-Normed Fit Index); CFI, Comparative Fit Index; GFI, Goodness of Fit Index; AGFI, Adjusted Goodness of Fit Index*.

### Empirical Results

#### Path Analysis

Table 6 shows the path coefficient analysis for verification of the causal relationship between variables. Positive implicit followership (PIF) (β = 0.374, *p* < 0.05) and negative implicit followership (NIF) (β = −0.326, *p* < 0.05) significantly impact on perceived supervisor support (PSS), therefore hypotheses 1 and 2 are supported. Perceived supervisor support (PSS) (β = 0.430, *p* < 0.05) significantly impacts self-efficacy (PSE), supporting hypothesis 3 of the research study.

**Table 6:** Path analysis.

**DV**	**IV**	**Unstd**	**S.E**.	**Unstd./S.E**.	***p*** **-value**	**Std**.	**R2**
PSS	PIF	0.570	0.092	6.214	0.000	0.374	0.299
	NIF	−0.389	0.077	−5.065	0.000	−0.326	
PSE	PSS	0.415	0.055	7.523	0.000	0.430	0.185

*PSS, Perceived supervisor support; PSE, Self-efficacy; PIF, Positive implicit followership; NIF, Negative implicit followership*.

#### Indirect Effect Analysis

Empirical studies considered utilizing bootstrapping mediation analysis is better than the B-K (Barron and Kenny's) approach or product of coefficient when evaluating indirect/mediation effects (MacKinnon et al., 2004; Williams and MacKinnon, 2008). Because the assumption of the normalized distribution of indirect effect can be ignored in the analysis, using bootstrapping mediation analysis has the advantage over the other two methods. When bootstrapping, the product coefficient of a and b is estimated for each sampling with replacement. The distribution of the product of a and b derives standard errors and confidence intervals. Five thousand times of sampling processes are recommended, 1,000 times at least (Hayes, 2009). Because bootstrapping mediation analysis can provide confidential intervals to examine the indirect effects, it is better than the other mediation testing methods. One of the preferable bootstrapping mediation analysis methods is bias-corrected bootstrapping (Briggs, 2006).

As shown in Table 7, the indirect effect PIFT → PSS → PSE, and NIFT → PSS → PSE were supported.

**Table 7:** Indirect effect analysis.

**Hypotheses**	**Path coefficients**	***T*** **-values**	***p*** **-values**
H4: PIF → PSS → PSE	0.314	3.056	0.002
H5: NIF → PSS → PSE	−0.152	−3.912	0.000

*PSS, Perceived supervisor support; PSE, Self-efficacy; PIF, Positive implicit followership; NIF, Negative implicit followership*.

#### Moderation Effect Analysis

Moderator (Mo) is an external variable, also called the interference variable, which can affect the relationship between the independent variable (X) and the dependent variable (Y). The relationship between the independent variable (X) and the dependent variable (Y) is usually determined by its slope. The moderator will affect the slope, the direction, and/or the strength of the relationship between the predictor variable and the criterion variable (Baron and Kenny, 1986). Moderator can be a categorical variable (e.g., age, gender, education), quantitative variable (e.g., satisfaction), latent variable (e.g., attitude), or observed variable (e.g., height, weight) (Busemeyer and Jones, 1983). Before the moderator can be introduced, the difference in the relationship between the independent variable (X) and the dependent variable (Y) must be computed.

Assuming linear relationships exist among independent variables, dependent variables, and the moderators, the moderating effect is computed by multiplying the independent variable and moderator (Busemeyer and Jones, 1983). If the multiplication of the independent variable and moderator has a significant impact on the dependent variable, moderating effect exists. Work friendship (WF) is a moderator in our proposed model. As shown in Table 8, the moderating effect of PSS^*^WF to PSE is 0.020 (z =|2.380|>1.96, *p* = 0.017). Since p<0.05, moderating effect exists (as shown in Table 8). For every 1 unit of the moderator (WF), the slope of PS to EF will increase by 0.020.

**Table 8:** Moderator effects analysis.

**DV**	**IV**	**Estimate**	**S.E**.	**Z-Value**	***p*** **-value**
PSE	PSS	0.038	0.045	0.856	0.392
	WF	0.197	0.021	9.526	0.000
	PSS*WF	0.020	0.008	2.380	0.017

## Discussion and Conclusion

This research attempts to explore its influence mechanism on new employees' self-efficacy from the perspective of followers' implicit followership. Effective onboarding can increase benefits by hiring talented employees and increase the utilization of the hard work spent in recruiting and selecting these employees (Smart, 2012). If implicit Followership is observed In the early stage of employees' career development, the new employee's work personality is not fixed. They are sensitive to the new environment. Therefore, the environment would impact new employees greater. The previous research believed that employees' lack of self-confidence and ability keep them from actively showing relevant behaviors (Haslam and Platow, 2001). If new employees can effectively improve their self-efficacy during the phase of onboarding of the organization, it will help eliminate the anxiety of new employees entering the organization and improve their work attitudes and behaviors (Gruman et al., 2006).

The research results show that the followers' positive implicit followership has a positive impact on the perceived supervisor support. On the other hand, the followers' negative implicit followership has a negative impact on the perceived supervisor support. This research result is similar to the research of Yang et al. (2020). Furthermore, similar research was conducted by Gao and Wu (2019) to study the association between IFT and employees' career success in 12 large corporations of China. In their study, they used social exchange theory and cognitive information processing theory to study the major research constructs. According to the results of their study, IFT was significantly related to employee's success. The survey data of this research shows that the average number of positive implicit followership items is 6.049, which is the highest among all dimensions. The average number of negative implicit followership items is 3.317, which is the lowest among all facets. It proved that most of the new employees have a relatively positive implicit followership cognition. This positive implicit followership cognition guided employees to implement active socialization strategies, thereby reducing the uncertainty of the new environment. Furthermore, one of the negative implicit followership items mentioned “It usually takes a long time to clarify the content of the work.” The standard deviation of the item reaches 2.044, which is the highest of all items. It indicated that the new employees have a great difference in opinions on this item. The reason may be that some new employees lack relevant work experience and skills. So, they spend more time in the new environment to complete the work. Other new employees didn't take long to clarify their work. They can quickly integrate into the new environment through observation and learning, acquire work-related skills, find the focus of the work, and complete the work in a short time.

In the analysis of the influence path of implicit followership on perceived supervisor support, the regression unstandardized coefficient of positive implicit followership is 0.570, and the regression unstandardized coefficient of negative implicit followership is −0.389. It shows that both positive implicit followership and negative implicit followership effects are strong. However, they are in the opposite direction, thus confirming the hypothesis of this study. The reason for analyzing this conclusion may be: when followers hold positive implicit followership, they believe that they should have a positive attitude and enthusiasm for work. This positive emotion makes them more optimistic in the organization and easier to explain the behavior of others from a positive perspective. On the contrary, when followers hold negative implicit followership, the followers think that things outside of work have nothing to do with them. It does not matter if the relationship with others is unfriendly. This negative emotion makes it easy for individuals to misunderstand and prejudice others.

Moreover, the perceived supervisor support has a significant positive effect on self-efficacy. This result is the same as the previous studies. For example, Afzal et al. (2019) found that the employee's perceived supervisor support positively affected employees' perception of self-efficacy, and the perceived supervisor support enhanced employees' confidence in their ability to complete work in the workplace. A recent study conducted in various organizations of China utilized self-efficacy as a moderator to analyze the relationship of perceived supervisor support for strengths use (PSSSU) and the original employee strengths use. According to the results, self-efficacy was found to positively moderate the relationship between PSSSU and strengths use. Moreover, it was also inferred that self-efficacy impacts the employees' perceived supervisor support for strength use and their actual strength use (Ding and Yu, 2020). Then, one of the perceived supervisor support items, "My supervisor encourages employees to develop new skills,” has the highest average score at 5.954, which shows that new employees can feel encouraged by their supervisors. Furthermore, supervisors are concerned more about new employees' work skills learning.

According to the results of this study, PSS has a mediation effect on the relationship between IFT and PSE. The results of this study are somewhat similar to a study conducted by Van Woerkom and Kroon (2020), according to their study PSS was used as a mediator between the relationship of performance appraisal and motivation to improve (MTI) performance. This study also examined the indirect moderation effect of workplace friendship on a relationship between PSS and PSE. The results indicated significant moderation effects of workplace friendship on the relationship. The results of this study are somewhat similar to the study conducted by Yu et al. (2021). They also used workplace friendship as a moderator. According to their results, workplace friendship was found to have significant moderation effects between the relationship of job insecurity and negative emotions, as well as job insecurity and extra-role behavior.

## Theoretical Contributions

This research mainly discusses the implicit follower of new employees and explores the results of its impacts. Based on the theory of social exchange and social relations, combined with self-efficacy, and perceived supervisory support, this study puts forward the research framework and related hypotheses.

For individuals, entering a new company to work requires recruits to demonstrate their job role abilities in a relatively short time and integrate into the organization's social network (Thomas and Meglich, 2019). But the speed and effectiveness of this socialization process depend on individual characteristics and organizational factors. Employees' positive implicit followership determines the state in which employees will integrate into the organization's work. Establishing a relationship with a direct supervisor has been proved to be a key active adjustment behavior in the socialization process of new employees (Kammeyer-Mueller et al., 2011). Therefore, it is very crucial to understand the influence of new employees' implicit followership perception on perceived supervisor behavior.

## Practical Contributions

The onboarding of new employees is a very critical event in their careers (Thomas and Meglich, 2019). Studies have shown that the employee turnover rate is the greatest in the initial few months of employment (Smith et al., 2012). According to the results of this study, it is suggested that the organization's socialization policy for new employees should be promoted in the following three directions, to enhance the perceived self-efficacy of new employees.

### Guide Employees to Establish Positive Implicit Followership Cognition

From the present study, the new employees' positive implicit followership cognition would positively impact their perception of supervisor support. Then, when a new employee has negative implicit followership, the cognition negatively affects his perceived supervisor support. Therefore, when hiring new employees, organizations should try their best to recruit employees with positive implicit followership cognition. Even if they do not have the corresponding special skills, for the time being, they would have better self-expectations to strengthen their self-efficacy. In addition, after new employees are hired, through training and publicity, employees are guided to cultivate positive employee traits and behaviors. More importantly, employees must internalize these traits and behaviors as their perception of the follower role.

### Provide Multi-Faceted Support for New Employees

The study found that the perceived supervisor support can effectively improve the self-efficacy of new employees. New employees may have less prejudice against specific supervisors than their long-term followers in the organization (Seele and Eberl, 2020). The supportive behaviors are more likely to be perceived by new employees. When individuals feel that their supervisors are supporting them at work, they would be capable of getting help in adversity (Kossek et al., 2011). Therefore, this study suggests that the company can provide support to the new employees in two ways. On the one hand, the organization provides more supportive policy assistance to new employees. Because the supervisor usually acts as the agent of the organization, the organization's support for employees will be regarded by the employees as the support of the supervisor, thereby enhancing their self-efficacy. On the other hand, the supervisors should create a supportive leadership atmosphere as much as possible. Since the supervisors have special resources and powers in the company, they usually have a broader vision than employees. Therefore, it is recommended that supervisors give new employees more attention and help to improve their self-efficacy.

### Create an Atmosphere of Communication Between Employees

This study has proved that high-level workplace friendship can enhance the positive impact of new employees' supervisor support on self-efficacy. The low-level workplace friendships would weaken the positive impact of supervisor support on self-efficacy. Feldman (1981) believed that group integration is one of the core elements for new employees for their socialization. New employees work with colleagues, trust each other, and solve work problems together are effective ways to incorporate into the organization. Therefore, it is recommended that business managers encourage employees to communicate with each other, create a harmonious atmosphere for communication, and help employees establish workplace friendships.

## Research Limitations and Future Directions

This research mainly takes new employees <3 years as the survey object for sampling. First, this study used a single dimension of supervisor support measurement. However, some studies have shown that the perceived supervisor support has multiple dimensions. Supervisors may provide emotional support and instrumental support in the workplace. Perceived supervisor support (PSS) was used as an individual construct in this study, hence for a complete and detailed investigation, future researchers are advised to use a multi-dimensional PSS construct and add several attitudinal and cognitive antecedents of PSS for future potential research. Therefore, future studies can take multi-dimensional supervisor support instruments. Secondly, this study uses cross-sectional data for research design instead of longitudinal research, which can produce time-oriented perspective results. Therefore, it is recommended that future researchers use the longitudinal intertemporal design for research. In addition, the subject of this research is mainly in Guangdong Province of China. Future research may select a wide range of subjects from other areas for research. Furthermore, China is an emerging country, therefore future researchers can target developed countries and compare the results.

## Data Availability Statement

The raw data supporting the conclusions of this article will be made available by the authors, without undue reservation.

## Author Contributions

WZ and X-JW contributed to research design, performed the sample collection, data analysis, conducted the research design, and wrote the manuscript. All authors have read and approved the final manuscript.

## Conflict of Interest

The authors declare that the research was conducted in the absence of any commercial or financial relationships that could be construed as a potential conflict of interest.

## Publisher's Note

All claims expressed in this article are solely those of the authors and do not necessarily represent those of their affiliated organizations, or those of the publisher, the editors and the reviewers. Any product that may be evaluated in this article, or claim that may be made by its manufacturer, is not guaranteed or endorsed by the publisher.
